# Supplementary figures and images for: Porphyrobacter mercurialis sp. nov., isolated from a stadium seat and emended description of the genus Porphyrobacter
Source: PeerJ. 2015 Nov 12;3:e1400. doi: 10.7717/peerj.1400 (PMC4647569; doi:10.7717/peerj.1400)

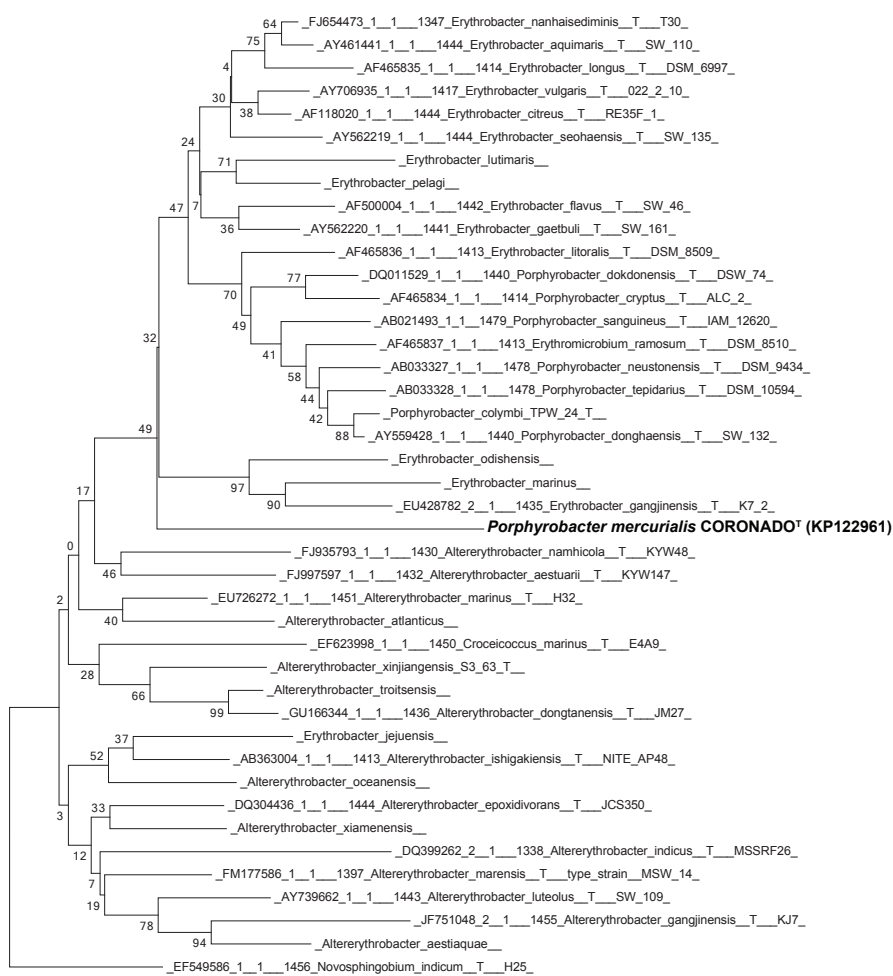

0.005

Supplement: Supplemental Information 1 — Tree was inferred using MEGA6. Numbers at nodes represent bootstrap values from 1,000 iterations of the tree. The tree was rooted to Novosphingobium indicum as an outgroup since this species was shown to be one of the closest relatives to the Erythrobacteraceae family in a tree of all Alphaproteobacteria. [file peerj-03-1400-s001.pdf]
